# Dysregulation of the Autonomous Nervous System in Patients with Temporomandibular Disorder: A Pupillometric Study

**DOI:** 10.1371/journal.pone.0045424

**Published:** 2012-09-18

**Authors:** Annalisa Monaco, Ruggero Cattaneo, Luca Mesin, Irma Ciarrocchi, Fabrizio Sgolastra, Davide Pietropaoli

**Affiliations:** 1 Department of Life, Health and Environmental Sciences, University of L’Aquila, L’Aquila, Italy; 2 Department of Electronics and Telecommunications, Politecnico di Torino, Torino, Italy; Tokyo Metropolitan Institute of Medical Science, Japan

## Abstract

The role of the autonomic nervous system (ANS) was recently investigated in Temporomandibular disorders (TMD). Several authors argue that in subjects with TMD there is a dysregulation of ANS. Recent literature support that Pupillometry is a simple non-invasive tool to study ANS. The aim of this study was to investigate the relationship between TMD and ANS activity using pupillometry recording in Infrared light at rest Mandible Position (RP); Infrared light at Forced Habitual Occlusion (FHO); Yellow-green light at RP; Yellow-green light at FHO. Forty female subjects were enrolled: 20 case patients showed TMD based on the Research Diagnostic Criteria for TMD, and 20 control patients, aged matched, had no signs or symptoms of TMD. Statistical analysis was performed on average pupil size. Ratio between pupil size in FHO and RP (FHO/RP ratio) and yellow-green and infrared (light/darkness ratio) lighting were carried out. Within group differences of pupil size and of “ratio” were analyzed using a paired t test, while differences of pupil size between groups were tested using an unpaired t test. Statistical comparisons between groups showed no significant differences of absolute values of pupil dimension in RP and FHO, both in yellow-green and in infrared lighting. In addition, there were no significant differences within groups comparing RP and FHO in yellow-green light. In within group comparison of pupil size, differences between RP and FHO were significant in infrared conditions. Control subjects increased, whereas TMD patients decreased pupil size at FHO in infrared lightening. FHO/RP ratio in darkness and light/darkness ratio in RP were significantly different between groups. Taken together, these data suggest that TMD subjects have an impairment of the sympathetic-adrenergic component of the ANS to be activated under stress. The present study provides preliminary pupillometric data confirming that adrenergic function is dysregulated in patients with TMD.

## Introduction

The role of the autonomic nervous system (ANS) was recently investigated in Temporomandibular Disorders (TMD). Several authors argue that in subjects with TMD there is a dysregulation of ANS [1], [2], [3]. Such a dysregulation is in part based on genetics [4]. It implies that the patients’ enhanced sympathetic drive inhibites normal catecholamine release resulting in significant effects on peripheral target organs and functions of the ANS, which would become less efficient in adapting to the needs of environmental and physiologic demands [5].

Some authors demonstrated the influence of the sympathetic branch of ANS on the muscles and dynamics of the jaw in animals and humans [6], [7], [8], [9], [10].

The control of contraction and dilation of the pupil is due to the sympathetic nerve centers (Budge’s ciliospinalis centre) and parasympathetic centre (Edinger- Wesphal Nucleus). The first promotes the pupil dilation (mydriasis), the second the constriction (miosis) according to the light stimulation.

Pupillometry is a simple and non-invasive tool to assess the size of the pupil. It is considered a reliable tool in the study of pupil effects of drugs [11], [12], [13] and literature supports that it is a valid method to study the ANS [14].

Pupil diameter significantly correlated with heart rate variability [15] and showed its usefulness for assessing the ANS dysregulation in clinical conditions in which the ANS is involved [16], [17], [18], [19], [20], [21].

The aim of this work is to study the effect of light condition and of dental occlusion on ANS behavior through the study of the pupil in subjects with TMD. The hypothesis is that these subjects exhibit a reduced ability to adapt and are less able than control subjects to modify the response of the pupil to activation induced by occlusal and light variations.

## Materials and Methods

### Subjects

This study was conducted in accordance with the Declaration of Helsinki. The Committee on Ethics in Science of the University of L’Aquila, L’Aquila, Italy approved the study and informed consent was obtained from each subject.

### Inclusion/Exclusion Criteria

20 patients that fulfilled the following criteria were included in the study: age less than 30 years; female gender; presence of complete permanent dentition, with the possible exception of the third molars; normal occlusion; and diagnosis of unilateral arthrogenous TMD, based on the Research Diagnostic Criteria for TMD (RDC/TMD) [22] Axis I, groups II and III in remission at least from 3 months.

Patients were excluded from the study if they met one or more of the following criteria: having systemic or metabolic diseases; eye diseases or visual defects; history of local or general trauma; neurological or psychiatric disorders; muscular diseases; cervical pain; bruxism, diagnosed by the presence of parafunctional facets and/or anamnesis of parafunctional tooth clenching and/or grinding; pregnancy; assumption of anti-inflammatory, analgesic, anti-depressant, opioid or myorelaxant drugs; smoking; fixed or removable prostheses; fixed restorations that affected the occlusal surfaces; either previous or concurrent orthodontic or orthognathic treatment.

Control Group consisted of 20 young women scheduled for a routine checkup at University Clinic, aged matched, without signs or symptoms of TMD, who fulfilled the inclusion and exclusion criteria.

### Pupillometry

Pupillometry was performed with a table-mounted infrared pupillometer (Oculus system, Inventis srl, Padova, Italy), which is composed by two infrared CCD cameras (resolution of 720×576 pixels, 256 grey levels) mounted on a light helmet (1.5 kg), with sampling frequency of 25 frame/s. To stabilize accommodation, the subjects were asked to focus their eyes on the light point into the pupillometer [23]. Assessment of pupil size was performed in light conditions, illuminating the eyes with a yellow-green led with 740 nanometer of wave-length, and under dark conditions that were obtained with an infrared diode with 880 nanometer of wave-length.

Pupillometric recordings were acquired in digital form and processed through the algorithm of Tarjan (of strongly connected components) [24] to measure frame by frame the area of pupil, expressed as number of pixels. A template was positioned on the computer screen allowing to correct the position of the eyes to avoid errors due to different positions of the pupil.

Pupillometry was performed with the subjects in horizontal supine position, on a bed for clinical examination. Room temperature (21°C) and relative humidity (50%) were maintained constant. Any external or internal source of noise was excluded. Before any pupillometric recording session, patients were invited to lie on an examining table with open eyes for at least 3 minutes, to adapt to the temperature and humidity of the room, as well to reduce the anxiety status. Then the pupillometer was applied and maintained until the end of the recording session.

### Recording Procedure

The subjects underwent to 4 subsequent recordings of 30 s each:

1. Infrared light at Rest Mandible Position (RP);
2. Infrared light at Forced Habitual Occlusion (FHO);
3. Yellow-green light at RP;
4. Yellow-green light at FHO.

The sequence of tests was assigned randomly. At the end of each test, a period of 1 minute followed in which subjects were asked to stay with eyes closed. Each new acquisition started 15 seconds after the opening of the eyes.

FHO was standardized by surface electromyography (SEMG). Disposable electrodes (Duotrode, bipolar surface electrodes Ag-AgCl, 20 mm center to center distance, Myotronics-Noromed, Inc., Tukwila WA, USA) were used for SEMG recording on right and left masseter. The electrodes were connected to SEMG equipment (K7/EMG, Myotronics-Noromed, Inc., Tukwila WA, USA). A pretest established, for each subject, the value of SEMG amplitude (Averaging) corresponding to maximum voluntary clenching. During FHO tests, SEMG values were maintained with verbal instructions from the operator between 30 and 50% of the maximum voluntary clenching.

### Statistical Analysis

Statistical analysis was performed using STATA 10 (StataCorp LP, College Station, TX, USA) on average pupil size, computed on 30 seconds of recording. Ratio between pupil size in FHO and RP (referred to as FHO/RP ratio in the following) and yellow-green and infrared (light/darkness ratio) lighting were carried out. Shapiro-Wilk test revealed normal distribution of data. Within group differences of pupil size and of “ratio” were analyzed using a paired t test, while differences of pupil size between groups were tested using an unpaired t test. The level of significance was set at p<0.01 for all tests. Results are expressed as mean and standard deviation.

## Results

Statistical comparisons between groups showed no significant differences of absolute values of pupil dimension in RP and FHO, both in yellow-green and in infrared lighting. In addition, there were no significant differences within groups comparing RP and FHO in yellow-green light.

In within group comparison of pupil size, differences between RP and FHO were significant in infrared conditions. In particular, in Control Group, pupil had significantly larger size (p = 0.005) in FHO compared to RP, while in TMD group the size of pupil was significantly lower (p = 0.009) in FHO compared to RP (Table 1).

**Table 1:** Pupil size (pixels) and statistical comparisons in different conditions in two groups.

	RP in yellow–greenlight condition	FHO in yellow-greenlight condition	RP in infrared lightcondition	FHO in infrared light condition
**Control Group**	4835.38 (1858.24)	4712.69 (1637.35)	7680.72 (2051.01)	8225.91* (1938.80)
**TMD Group**	4289.51 (1543.59)	4258.83 (1583.17)	8412.75 (1956.50)	8199.52** (1956.51)
**t Test**	0.21	0.24	0.18	0.48

In brackets the standard deviation. * Comparison between RP and FHO condition in infrared light within Control Group: p = 0.005. ** Comparison between RP and infrared light in FHO condition within TMD Group: p = 0.009.

FHO/RP ratio did not present significant differences in yellow-green light conditions in between groups comparison, whereas in infrared light condition the comparison between groups showed a highly significant difference (p = 0.0006) with a larger ratio in Control Group (1.055) compared to TMD Group (0.967) (Table 2).

**Table 2:** FHO and RP ratio in Control and TMD Group.

	Yellow-green light	Infrared light
**FHO/RP ratio Control Group**	0.996 (0.149)	1.055 (0.073)
**FHO/RP ratio TMD Group**	1.002 (0.169)	0.967 (0.037)
**t Test**	0.46	0.0006

Statistical comparison between the two groups. Significance level at p = 0.01. In brackets the standard deviation.

Light/darkness ratio showed a high significance (p = 0.0008) in the comparison between Control Group (0.662) and TMD Group (0.486) at RP, while there was no significant difference between the two groups at FHO (Table 3).

**Table 3:** Light/darkness ratio at RP and FHO.

	RP	FHO
**Light/Darkness ratio Control Group**	0.662 (.099)	0.545 (0.345)
**Light/Darkness ratio TMD Group**	0.486 (0.137)	0.510 (0.375)
**t Test**	0.0008	0.24

Comparison between Groups. Significance level at p = 0.01. In brackets the standard deviation.

**Figure 1:**
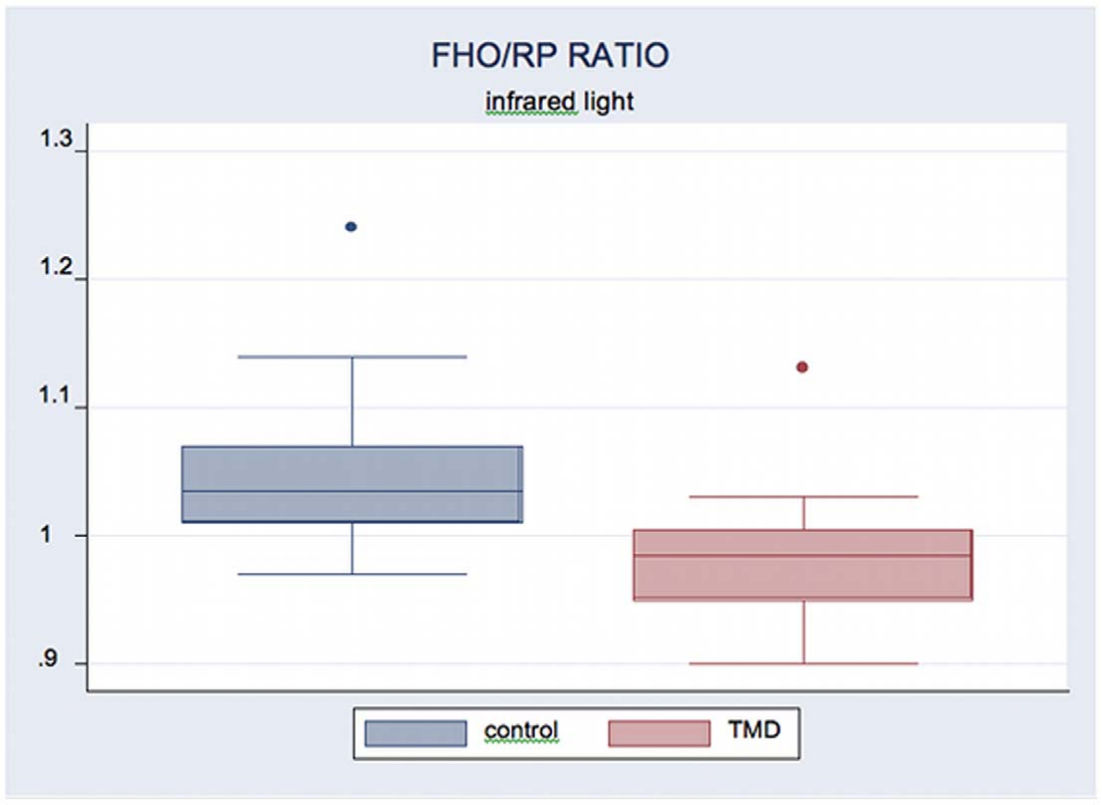
Box plot of FHO/RP ratio in infrared light condition.

**Figure 2:**
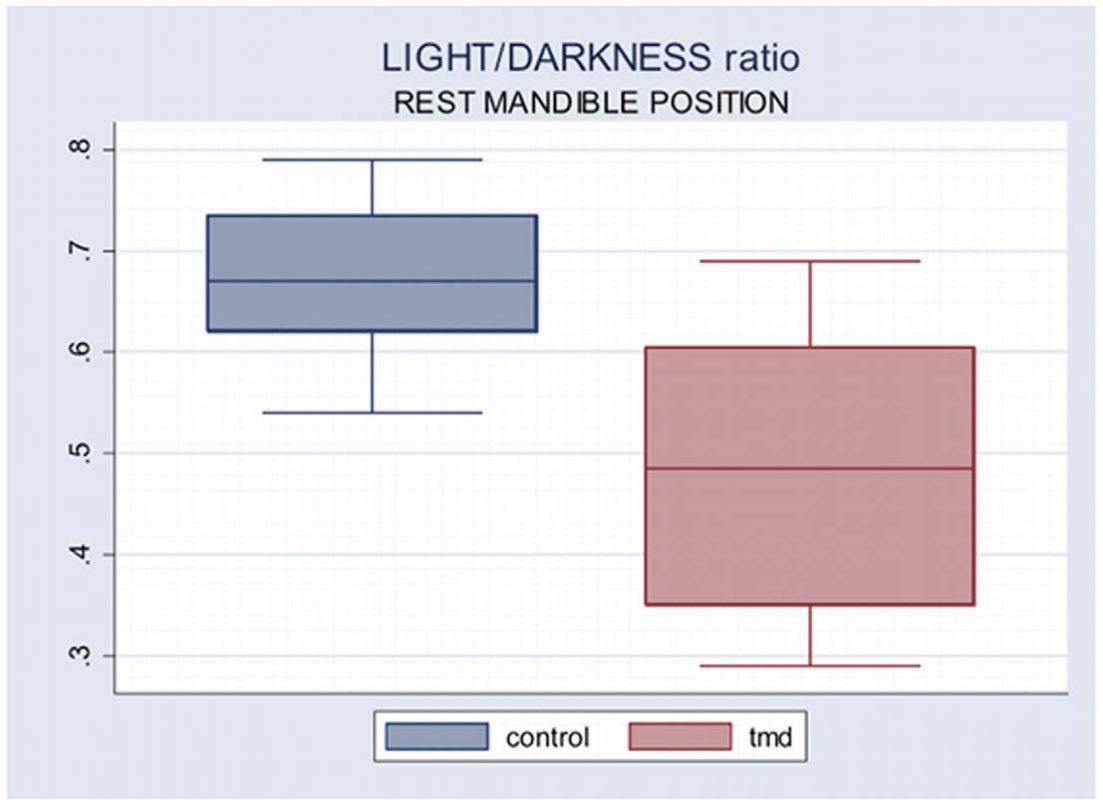
Box plot of Light/Darkness ratio in rest mandible position.

Figures 1 and 2 show box plots of the distribution of FHO/RP ratio in infrared light and light/darkness ratio at RP.

## Discussion

The present study provides preliminary pupillometric data confirming ANS dysregulation in TMD patients.

In particular it should be noted that: a) TMD patients show values of pupil size greater in RP infrared and lower in RP light condition than control subjects, although not statistically significant; b) under stress condition (light-FHO) TMD subjects behave differently compared to control subjects: FHO/RP ratio in darkness and light/darkness ratio in RP have significantly different behavior in the two groups. Control subjects increased, whereas TMD patients decreased pupil size at FHO in infrared lightening. In infrared conditions FHO/RP ratio is significantly greater in Control Group as well as light/darkness ratio at RP.

Pupil size in RP infrared is greater in control than in TMD group. The comparison between groups does not indicate statistical significance, probably because of dispersion of values.

On the other hand, comparison within group at infrared lighting shows that FHO pupil size of the control subjects increases significantly compared to RP, while FHO pupil size of TMD subjects decreases significantly.

Difference of pupil behavior between groups, highlighted by the ratio between the two different conditions (FHO/RP), becomes significant because in control group it is directed toward a ratio greater than 1 (1.055) for the increase of the size in FHO respect to RP, while in the TMD group it is directed toward a ratio less than 1 (0.967) for the reduction of the size in FHO compared to RP.

Homogeneous behavior of FHO/RP ratio within group was highlighted by the low standard deviation, especially in FHO infrared condition (0.0734 in control group and 0,037 in TMD group).

Similar argument can be made for RP light/darkness ratio higher in control than TMD subjects. In RP infrared, as above discussed, the pupil size is lower, although not significantly, in control compared to TMD subject (7680.72 vs 8412.75), while in RP yellow-green light the pupil size is greater in control than in TMD subjects (4835.38 vs 4289.51).

As a speculative, it can be suggested that TMD subjects show a greater activation or less inhibition of iris muscles contraction under conditions of specific stimulation (presence or absence of light) and less activation or greater inhibition of the contraction under generic stimulation (FHO).

In any case, certain degree of dysregulation would occur between the two branches of ANS, suggesting that TMD subjects could suffer from impairment to be activated under stress.

Pupil size is controlled by complex interaction between sympathetic and parasympathetic branches of ANS: the first uses mainly adrenergic, the second cholinergic pathway.

Both muscles of iris (sphincter and dilator) receive reciprocal innervations from the 2 branches of ANS providing contraction and inhibition (or relaxation).

Parasympathetic cholinergic fibers, coming from Edinger-Wesphal Nucleus, supply the iris sphincter acting for contraction of the muscle and the consequent reduction of the pupil size. At the same time, the sphincter receives beta-adrenergic innervations able to reduce the contraction inducing relaxation of the muscle [43], [44]. In humans pupil dilation obtained by beta-adrenergic inhibition of sphincter can be equal to 1/3 of the maximum physiological dilation [45].

On the other hand, iris dilator muscle receives a predominant adrenergic sympathetic motor innervation (Budge’s Cilio Spinal Center) causing contraction and the consequent increase in pupil size. Dilator muscle contraction is mediated by alpha-adrenergic receptors and inhibition or relaxation may be exercised by muscarinic receptors and, although not yet fully documented, by beta-adrenergic innervations [46], [47], [48], [49], [50].

The consequence of dysregutated balance of reciprocal innervations may lead to a deficit of inhibitory effect. Defect in beta-adrenergic or muscarinic inhibition that counteracts the action of alpha-adrenergic contraction of the dilator may result in increased pupil size in darkness; at the same time, reduction of beta-adrenergic inhibition on cholinergic system of sphincter muscle could lead to increase of miosis.

Etiology of TMD remains somewhat uncertain. Some authors have shifted the focus away from stomatognathic system and occlusion suggesting that ANS could play a crucial role in generating the main signs and symptoms of this disorder [1], [2], [3], [51].

Dysregulation of ANS and the phenomena associated with chronic cortisol secretion [25], [26] would be crucial in the pathogenesis of TMD. Causes of TMD related to type of occlusion or masticatory muscle hyperactivity did not give conclusive results. Beyond some partial results, occlusion or muscular activity cannot be surely assumed at the base of TMD. Muscles, however, could be called into question secondarily by presence of ANS dysregulation with regard to coordination of simple and complex functions [6], [7], [8], [9], [10].

On the other hand, some TMD symptoms can be reduced significantly by pharmacological therapies aimed at improving the sympathetic-adrenergic regulation. Beta-adrenergic system seems to be particularly involved. TMD patients showed lower plasma epinephrine (EPI) and norepinephrine (NE) levels than healthy subjects [5]. Moreover, several studies have demonstrated the effect of propranolol (sympatholytic non-selective beta blocker) on clinical pain severity showing linear relationship between beta-adrenergic dysregulation and pain reduction after propranolol administration in genetic subgroup of TMD patients [27], [28], [52], [53].

Taken together, these works suggest that beta-adrenergic system could be one of the key dysfunction associated with TMD pain and its therapy.

As mentioned above, beta-adrenergic receptors could have an inhibitory role on dilator and sphincter muscle. In dilator muscle beta-adrenergic and muscarinic receptors could cooperate to mediate relaxation counterbalancing pupil dilation; in sphincter muscle beta-adrenergic receptors could counterbalance pupil constriction.

Our data seem in agreement with above observations about ANS dysregulation in TMD.

Beta-adrenergic system of iris muscles in TMD patients could result in reduction or inhibition of the counterbalancing action on alpha-adrenergic pupil dilation system; the effect could be the increase of pupil size in darkness, whereas beta-adrenergic defect on cholinergic sphincter contraction, determining reduction of inhibitory effect, could result in greater miosis in yellow-green lighting. The light/darkness ratio smaller in TMD subjects compared with control group could be explained in the same way.

In control healthy group and in conditions of darkness, during which the tonus of sympathetic system prevails, occlusal stress produces a further increase of pupil size, whereas in TMD subjects occlusal “stress” fails to obtain the same sympathetic activation. TMD subjects showed within group significant reduction in pupil size. It could be interpreted as a failure of the sympathetic response to stress.

FHO can be compared to muscle exercise involving activation of the sympathetic system and a simultaneous variation of the pupil size [29], [30]. Clenching, in fact, produces sympathetic activation, partly via an increase of blood flow and pressure of the head [31], [32], [33].

Accordingly, in our work the size of pupil increases during clenching in control group showing ANS response to the increase of the demand. In subjects with TMD this phenomenon is not present. In these subjects, the size of pupil decreases. Effect of type of dental occlusion on results could be excluded by strict inclusion criteria.

On the other hand, in RP yellow-green lighting TMD subjects show more accentuated reaction compared to control subjects. TMD subjects’ pupil is smaller in light condition and is greater in darkness than controls’ one. Consequently, the RP light/darkness ratio is lower in TMD compared to control subjects. This could also be in accordance with the above-discussed deficit of beta-adrenergic inhibitory control of the iris sphincter muscle.

In general, pupil size reduction can be determined by light, parasympathomimetics substances, relaxation and convergence of the eyes. All mentioned phenomena have been checked through the experimental set up and no substances (sympathetic or parasympathomimetic) were taken by the subjects during the experimental session.

The degree of relaxation could be different in control and TMD subjects, in particular the latter may have difficulty to adapt to experimental conditions in relation to psychological characteristics described in literature [34], [35], [36].

Mood disorders, frequently related to TMD, involve dysregulation of the sympathetic system [37], [38], [39], [40], [41]. Some evidences stated that pupil diameter in the dark is not affected by anxiety but responses to light stimuli in the anxious patient had consistently lower amplitudes [42], suggesting a greater supranuclear inhibition of the parasympathetic oculomotor reflex arc in the anxious patients.

In our work we studied tonic pupil behavior in constant lightening (yellow-green and infrared) and we don’t have any direct information about pupil reflex. For this reason it’s impossible to compare our results (lower light/darkness ratio, lower FHO/RP ratio in infrared light, lower pupil size in yellow-green light, greater pupil size in darkness of TMD group) apparently in disagreement with above cited [42], [50] using light reflex. Moreover, we didn’t use psychometric scales to measure mood and psychological characteristics of subjects. We can’t exclude psychological influence on our TMD and control sample.

Our data show altered tonic adjustments of ANS in TMD patients. On the other hand, since the sequence of tests was randomized it is unlikely that the effects observed in the study are strictly related to environmental or emotional adaptation.

Of course, the interpretation of our results need more data, the sample is too small to draw definitive conclusions. Our study used pupillometry to monitor the activity of ANS without comparing pupillometric data to other data coming from signals already used in literature to study ANS (HRV, electrodermal activity, etc.). Probably, this is one reason why we can only suggest a speculative interpretation of data. Next work will be devoted to confirm what suggested interfacing various signals and sampling specific population, for example comparing TMD patients treated with beta blockade drugs with untreated.

This work can be considered a pilot study. If confirmed, it could provide a useful, rapid and noninvasive tool to test ANS behavior in TMD patients, and to monitor the response to therapy quickly and directly in dental office.
